# Diagnostic performance of NxTek^™^ Eliminate Malaria-Pf test for the detection of *Plasmodium falciparum* in school children with asymptomatic malaria

**DOI:** 10.1186/s12936-023-04529-y

**Published:** 2023-03-29

**Authors:** Abdissa Biruksew, Ashenafi Demeke, Zewdie Birhanu, Estifanos Kebede, Lemu Golassa, Evans Mantiri Mathebula, Delenasaw Yewhalaw

**Affiliations:** 1 School of Medical Laboratory Sciences, Faculty of Health Sciences, Institute of Health, Jimma University, Jimma, Ethiopia; 2 Tropical and Infectious Diseases Research Center (TIRC), Jimma University, Jimma, Ethiopia; 3 Department of Health, Behavior, and Society, Faculty of Public Health, Institute of Health Jimma University, Jimma, Ethiopia; 4 Arba Minch Health Science College, Arba Minch, Ethiopia; 5 Aklilu Lemma Institute of Pathobiology, Addis Ababa University, Addis Ababa, Ethiopia; 6 School of Health Systems and Public Health, Faculty of Health Science, University of Pretoria, Pretoria, South Africa

**Keywords:** NxTek^™^ Eliminate Ag Pf, hsRDT, qPCR, Asymptomatic falciparum malaria, School children, Ethiopia

## Abstract

**Background:**

One of the major roadblocks to the falciparum malaria elimination programme is the presence of a portion of the population, such as school children, with asymptomatic malaria infection. Targeting such reservoirs of infections is critical to interrupting transmission and enhancing elimination efforts. The NxTek^™^ Eliminate Malaria Pf test is a highly sensitive rapid diagnostic test (hsRDT) for the detection of HRP-2. However, knowledge gaps exist in Ethiopia on the diagnostic performance of hsRDT for the detection of *Plasmodium falciparum* in school children with asymptomatic malaria.

**Methods:**

A school-based cross-sectional study was conducted from September 2021 to January 2022 on 994 healthy school children (aged 6–15 years). Finger-pricked whole blood samples were collected for microscopy, hsRDT, conventional RDT (cRDT or SD Bioline Malaria Ag Pf/P.v), and QuantStudio^™^ 3 Real—Time PCR system (qPCR). The hsRDT was compared to cRDT and microscopy. qPCR and microscopy were used as reference methods.

**Results:**

The prevalence of *Plasmodium falciparum* was 1.51%, 2.2%. 2.2% and 4.52%, by microscopy, hsRDT, cRDT and qPCR, respectively. Using qPCR as reference, the sensitivity of hsRDT was higher (48.89%) than the microscopy (33.3%), and showed 100% specificity and a positive predictive value (PPV). Microscopy showed similar specificity and PPV as hsRDT. Using microscopy as a reference, the diagnostic perforrmances of both hsRDT and cRDT were similar. Both RDTs demonstrated identical diagnostic performances in both comparison methods.

**Conclusions:**

hsRDT has the same diagnostic performance as cRDT but improved diagnostic characteristics than microscopy for detection of *P. falciparum* in school children with asymptomatic malaria. It can be a useful tool for the national malaria elimination plan of Ethiopia.

## Background

Malaria continues to be a major public health problem putting nearly half of the world's population at risk despite years of significant rollback efforts [1, 2]. A total of 241 million cases were reported from 85 endemic countries in 2020, the number of cases rose to further 14 million with mortality rose from 534,000 to 602,000 compared to 2019, with the most dominant species of these cases being attributed to *Plasmodium falciparum* [1].

In Ethiopia, malaria is a major public health problem with approximately 60% of the population is at risk [3]. The peak transmission occurs from September to December after the main rainy season (June–August), while the minor one is from March to June every year after a rain shower, and is typically endemic below an altitude of 2000 m above sea level. As a result, it is often characterized by localized and broader epidemic cycles [4–6].

Over the past decade, Ethiopia has committed to strengthened malaria control efforts and significant progress has been made in reducing malaria-related morbidity and mortality. In line with the Global Technical Strategy on Malaria 2016–2030 (GTS), the country adopted a National Malaria Elimination Roadmap (later launched as National Malaria Strategic Plan 2021–2025) in 2016, envisaging a malaria-free Ethiopia by 2030 [3, 7]. To achieve the vision, the country’s key elimination strategies are to use conventional malaria diagnostic tools such as microscopy and malaria rapid diagnostic tests (RDTs), vector control, case treatment with artemisinin-based combination therapy (ACT), and use of a gametocytocidal agent, such as a single dose of primaquine [3]. However, a significant portion of the population is infected with *P. falciparum* malaria parasite, but is asymptomatic and is, therefore, invisible to the healthcare system and serves as a source of persistent transmission [8]. Among this segment of the population are a large number of asymptomatic school-age children, who have not received much attention and have benefited the least from universal malaria intervention [4–6]. It is one of the major challenges facing the national malaria eradication programme. The identification and targeting of such transmission reservoirs have major implications for the program. Currently being used successfully for clinical malaria diagnosis and immediate treatment, but are inherently unable to detect such a large population, which is suboptimal, allowing for sustained transmission of the disease to mosquitoes. For example, the limit of detection (LOD) for microscopy is 50–100 parasites/µL blood, reportedly missing 20–50% of infected individuals [9], while most conventional cRDTs fail to detect malaria when parasitaemia falls below the LOD 100–200 parasites/µL in asymptomatic carriers [10], leading to misdiagnosis and clinical decisions.

On the other hand, among the highly sensitive molecular tools for detecting low-density parasitaemias include the loop-mediated isothermal amplification method (LAMP) [11], conventional polymerase chain reaction (PCR), nested PCR and multiplex real-time PCR [12]. They are also important tools for detecting *P. falciparum* in areas where HRP2/3-based malaria RDTs are reported to be circumvented [13–15]. Unfortunately, these tools are expensive, require highly skilled personnel and a continuous power supply [16], and are only operated in research settings [12].

Therefore, there is an urgent need for a malaria RDT that is highly sensitive to detect low-density parasitaemias, quality-assured, and easy to use in remote settings with limited resources [^17^, ^18^]. Recent scientific recommendations also underlined the need to use such RDTs to identify asymptomatic infection reservoirs to achieve the goal of malaria elimination [19]. In this regard, the NxTek^™^ Eliminate Malaria Ag Pf [formerly Alere ultrasensitive Malaria Ag Pf RDT/Abbott Diagnostics Inc., Republic of Korea (05FK140)], is an hsRDT developed to detect *P. falciparum* HRP2 [20] in low-density infections. It has been prequalified by the World Health Organization (WHO), confirming its performance in detecting clinical malaria infections (parasitaemia > 200 parasites/µL) and the evaluation of this kit in population with asymptomatic malaria has not been validated [21]. A systematic review was conducted on its field performance revealing the hsRDT had higher sensitivity than cRTDs [^22^]. Few studies conducted field evaluation of the kit in Uganda and Myanmar in detecting HRP2 among malaria infected individuals including asymptomatic population in varying geographical settings showed superior sensitivity of the hsRDT over conventional malaria diagnostic tools [^20^, ^22^, ^23^].

In the Gomma District of the Jimma Zone in Ethiopia, microscopy and SD Bioline Malaria Ag Pf (HRP2/ Pv pLDH) are the routinely used malaria diagnostic tools. In Ethiopia, hsRDT has never been used as malaria diagnostic tool and data on the extent of asymptomatic malaria in school children is scarce. So far, no work has been published on the diagnostic performance of the hsRDT in detecting *P. falciparum* in school children with asymptomatic malaria in Ethiopia.

This study aimed to evaluate the diagnostic performance of the NxTek^™^ Elimination Malaria Pf Ag in detecting *P. falciparum* malaria in asymptomatic school children in Gomma District, Jimma Zone, Ethiopia.

## Methods

### Study setting and design

**Fig. 1:**
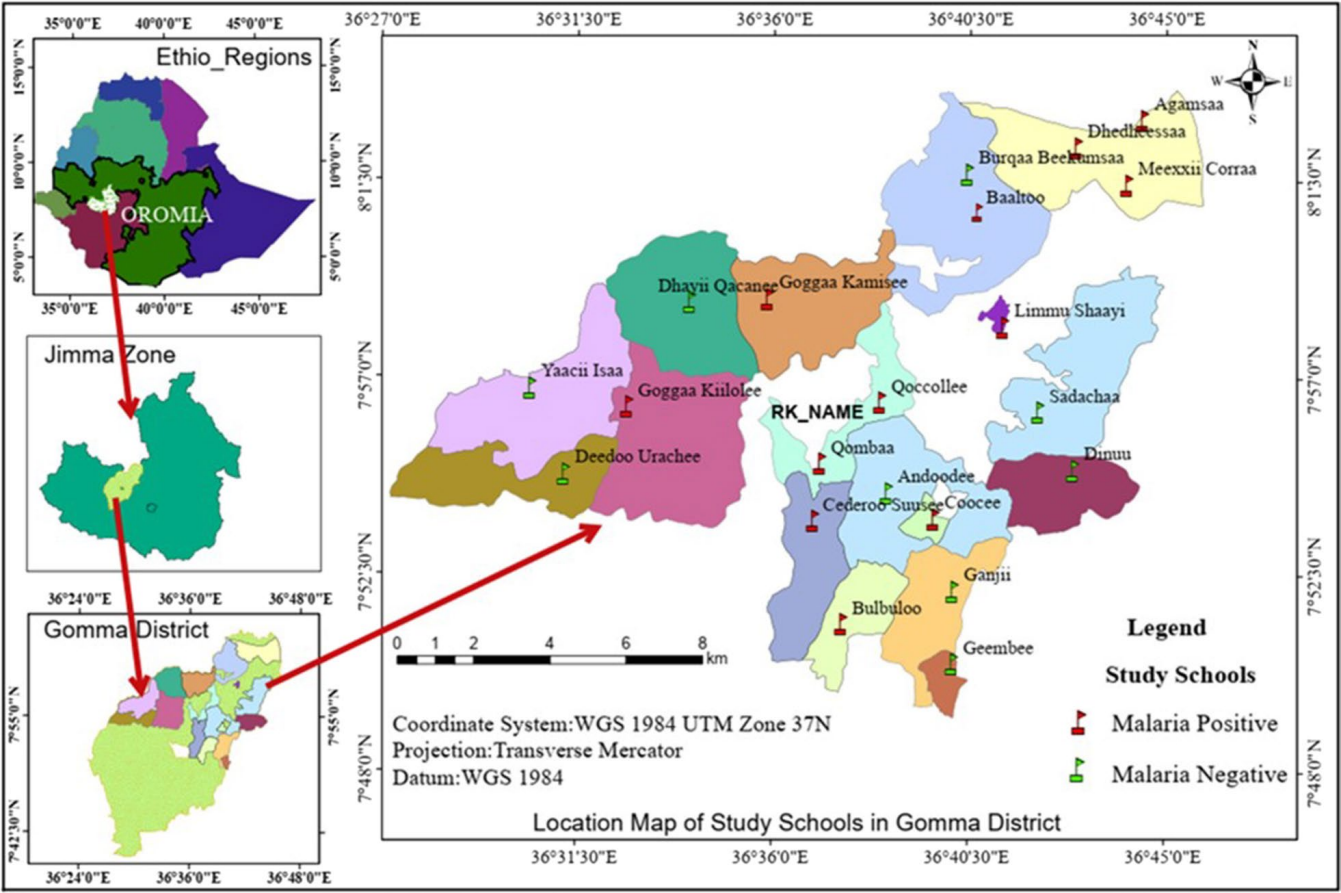
Map of study area and schools in Gomma district, Jimma Zone

A school-based cross-sectional study was conducted as part of the main project entitled “Asymptomatic malaria and its determinants in school children in Gomma district, Jimma zone, Oromia, Ethiopia”. The study site was 395 km southwest of Addis Ababa, Ethiopia and about 45 km west of Jimma town, the capital of the Jimma Zone (Fig. 1). It is also located at 7 49 59.99 north latitude and 36 39 59.99 east longitude and at an altitude of 1,380–1,680 m above sea level. The district consists of 42 administrative villages (known as Ganda, the lowest administrative level). Of the 80 schools in the district, five are high schools (9–12 grade) and 75 are primary schools (1–8 grade). There are 49 health facilities in the district. There are also 86 active health workers in the district [Jimma Zone Health Department, unpublished data]. The district receives rain all year-round and is endemic to malaria, primarily caused by *P. falciparum* (unpublished, Jimma Zone Health Department unpublished data). Malaria is high immediately after the main rainy season and then gradually declines thereafter. Only two *Plasmodium* parasite species are prevalent in the area, with the dominant species *P. falciparum* followed by *Plasmodium vivax*. Malaria prevention and control in the district is active. According to malaria stratification of the country [7], Gomma is classified as an area of low malaria transmission based on the Annual Parasite Incidence (API), i.e., API > 5 & < 10. Depending on the parasite species, age and situation of the patient, primaquine is administered as a radical cure and transmission blocking, respectively.

### Sample size and sampling technique

The sample size was calculated based on single population proportion formula:

$$\mathrm{n}=\frac{{\left(\mathrm{z}\frac{\mathrm{\alpha }}{ 2}\right)}^{2 }\mathrm{p}\left(1-\mathrm{p}\right)}{{\mathrm{d}}^{2}}$$

where n = the sample size, z = 1.96 at 95% Confidence Interval (CI), d is the margin of error at 2% (to maximize sample size since asymptomatic malaria is a rare infection), p is the expected prevalence rate for falciparum malaria among asymptomatic school children in the district was 6.8% [23]. Therefore, the calculated sample size (n) became 608. A non-response rate of 10% was added, resulting in 668. Finally, a design effect of 1.5 was applied to estimate the total sample of 1002 school children included in this study. In this study, multi-stage sampling technique was used. That is, Gomma district was randomly selected as the primary sampling unit from the list of 21 districts in Jimma zone. Of the 75 primary schools (1st through 8th grade), five were not accessible, so 21 were also selected as secondary sampling units from the list of 70 schools. The sample size was then distributed proportionally to the individual primary schools and then to the individual grades and sections. Finally, each school child was selected as the final sampling unit using the lottery method from lists of student records (rosters) and then stratified by age (6–15 years) and grade level (1–4 and 5–8) according to methods described by Brooker et al*.* [24] and previous work [23, 25].

### Inclusion and exclusion criteria

A school child aged 6–15 years whose family/guardian consented for and was not taking anti-malarial medication and had an auxiliary body temperature of  < 37.5 °C included in the study. School children who were on anti-malarial medication within 1 month of recruitment, had a fever at the time of data collection, and were older than 15 years were excluded from the study.

### Demographic data and blood sample collection

Following collecting demographic data (age sex, grade level and place of residency), finger-pricked whole blood samples were obtained from each study participant at each study school. After labeling with each participant's identification code (ID), 5 µL of blood was used for field detection of *P. falciparum* with hsRDT and cRDT and testing was performed according to the manufacturer’s instructions [26]. For microscopic examination, a labelled slide with frosted ends was used for both thick and thin blood smear preparation, air-dried and alcohol-fixed, and transported to the Medical Parasitology Laboratory at Jimma University, Institute of Health. Four drops of blood were also collected on Watmann filter paper-2 as dried blood spots (DBS), and stored at −20 °C in individual sterile plastic bags for PCR analysis.

### Microscopic examination

Blood smears were stained with 10% buffer diluted Giemsa for 10 min and examined through a 100X oil immersion objective. Species identification and parasite density were determined after slide reading by experienced laboratory technologists (who have experience in malaria microscopy as senior technicians at Jimma University), and each negative slide was declared after 100 fields were thoroughly examined [26]. Asexual blood stage parasite counts (density) were determined from a thick blood smear using the standard WHO formula [27] as follows:

$$\mathrm{Parasites\,}/\mathrm{ \mu L}\mathrm{\,of\,}\mathrm{ blood}=\frac{No\,of\,asexual\,parasite\,couted*8000\, White\, Blood\, Cells/\mathrm{\mu L}}{No\, of\, White\, Blood\, Cells\, counted}$$

**Fig. 2:**
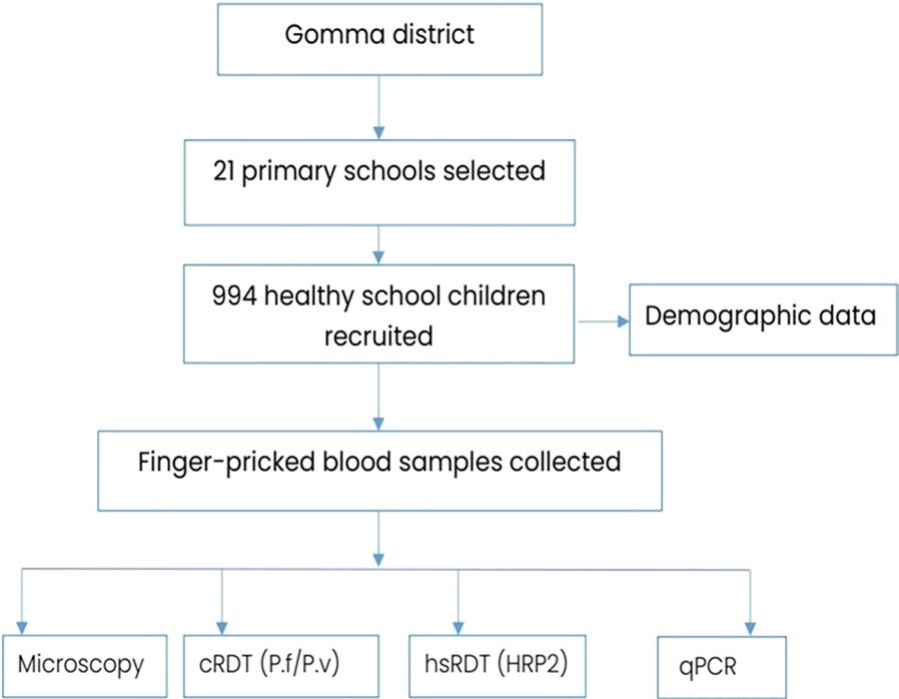
Study flowchar of diagnostic perormance of hsRDT in school children with asymptoatic malaria in Jimma zine, Southern Oromoa,Ethiopia

That is, the parasite count was performed against 500 white blood cell (WBC) counts, assuming a mean WBC count of 8,000/L. Quality control was carried out at every step of the process. Each blood smear was read independently by two experienced microscopists, each blinded to the other reader's results. Discordant results were resolved by a third expert microscopist who was blinded to the readings of the first two microscopists. And all the data collectors took training to ensure quality of the data as the study flowchart was followed (Fig. 2).

### NxTek^™^ Eliminate Malaria Pf Ag test (hsRDT)

NxTek Eliminate Malaria Pf is an hsRDT (Product Code 05FK140, Lot No. 05LDF009F, Expiry Date 22 Apr 01, manufactured by Abbott Diagnostics Korea Inc.) [21], a two-band test and a qualitative and differential test for the detection of *P. falciparum* HRP2 in human whole blood. 

### The conventional RDT (cRDT)

SD Bioline Malaria Ag (Pf- HRP2 and Pv-pLDH) is a triple band (one for *P. falciparum HRP*2, one for *P. vivax* pLDH and a control band), manufactured by Standard Diagnostics Inc. (now Abbot Diagnostic Korea Inc.), LOT number: 05DDF011A and product code 05fk80, Korea), developed for routine diagnosis of both *P. falciparum HRP*2 and pLDH of *P. vivax,* was used as a comparator. The test provides results within 15 min and is routinely used in remote healthcare facilities in Jimma zone, Ethiopia.

### The molecular assay

Approximately 3–5 mm diameter pieces of DBS were cut with a round hole puncher into a labeled 1.5 ml Eppendorf tube. Parasite DNA was then extracted from DBS using the Chelex100 resin saponin method as previously described [28] and the purified DNA was then transferred to Nunc tubes (0.5 mL) and stored at −20 °C until used for the PCR assay**.** For this PCR assay, the TaqMan probe for species-specific detection of *Plasmodium* parasite was used. QuantStudio^™^ 3 Real-Time Multiplex PCR system (Thermo Fisher Scientific) was operated to amplify the 18S rRNA genes for detection of *P. falciparum* and *P. vivax* using a pair of forward and reverse primer sequences and probes. For *P. falciparum* specific primers (5’-3’) [29]: F-F (forward): TATTGCTTTTGAGAGGTTTTGTTACTTTG and.

F-R (Revers): ACCTCTGACATCTGAATACGAATGC. And probe used was: Pf-fam (MGB): ACGGGTAGTCATGATTGAGTT.

For *P. vivax* specific primers used [30] was: Pv-1 (forward): CGCTTCTAGCTTAATCCACATAACTG, and Pv-2 (Reverse): AATTTACTCAAAGTAACAAGGACTTCCAAG.

And the probe used was: Pv-probe (VIC-MGB): CGCATTTTGCTATTATGT. For quality control, DNA from P. *falciparum* and P. vivax isolates (Pf and Pv MR4(BEI)) were used as positive controls, while molecular grade water was used as a negative control. In brief, PerfeCTa^®^ qPCR ToughMix^®^ (Low ROX^™^, Quanta Bio vwr, catalog number: 97065–968) was used as qPCR master mix. Briefly, PCR amplification was performed in a total reaction volume of 12 μL which contained 6 μL of PerfeCTa master mix, 0.5 μL(× 2) each of probe for *P. falciparum* (Pf-Fam) and *P. vivax* (Pv-vic), 0.4 μL(× 4) each of the forward and reverse primers, 2 μL extracted DNA and 1.4 molecular grade water under the PCR cycling conditions for 45 cycles. The initial denaturation was 50 ºC for 2 min, followed by 45 cycles of amplification at 95 ºC for 2 min, 95 ºC for 2 s and 60 ºC for 30 s with the total run-time of 60 min.

### Data analysis

Data were entered using Epi-data version 3.1, exported to Statistical Package for Social Sciences (SPSS) version 26, and analysed. The sensitivity, specificity, positive predictive value (PPV), negative predictive value (NPV) and accuracy were first determined against microscopy. This because of microscopy is the gold standard for malaria diagnosis. Then, against the qPCR due to its higher sensitivity to detect low-density parasitaemia. In all parameters, 95% CI was taken. Kappa values were used to assess the agreement between malaria diagnostic methods. The Chi-Square test was used to assess the association between characteristics of children and the positivity of each malaria diagnostic tool. A p-value of 0.05 was considered statistically significant.

### Ethics statement

Ethical approval was obtained from the Jimma University Institute of Health Research and Ethical Review Committee/Internal Review Board (IRB) (Ref: IHRPED/550/2). In addition, letters of permission were obtained from the Gomma District Health and Education Departments and presented for selected primary schools and local health departments. Information on the purpose, potential risks and benefits of the study was well addressed to each school child's family/guardian and presented in their own language. Fingerstick blood samples were taken after obtaining written informed consent from the parents or legal guardians of each school child. All malaria-positive children were treated at a nearby health facilities in accordance with the Federal Ministry of Health's malaria treatment guidelines after sending cover letters stating their name, age, gender, school and grade level. Confidentiality of each study participant’s data was maintained throughout this project.

## Results

### Characteristics of the study participants

A total of 1002 healthy school children included in the study and eight were excluded after field works based on exclusion criteria (for anti-malarial drug treatment), resulting in a total of 994 participants with normal axillary body temperature (< 37.5 °C) completing the study with the response rate is 99.2%. Of 994 school children who completed the study, as presented in Table 1, 52.4% (521/994) of them were males and 47.6% (473/994) were females with a mean age of 11.54 (± 2.5 std). The majority of students (77%) were from rural areas, while 23% were from semi-urban areas.

**Table 1:** Malaria diagnostic tools and characteristics of the participants

Characteristics		Total No	Malaria diagnostic tools							
			Microscopy		hsRDT (pfHRP2)		cRDT (PfHRP2)		qPCR	
			+ ve	−ve	+ ve	−ve	+ ve	−ve	+ ve	−ve
		994	15	979	22	972	22	972	45	949
Sex	Male	521 (52.40)	12 (80.00)	509 (52.00)	18 (82.00)	503 (51.70)	18 (82.00)	503 (51.70)	29 (64.40)	492 (51.84)
	Female	473 (47.60)	3 (20)	470 (48.00)	4 (18.00)	469 (48.30)	4 (18.00)	469 (48.30)	16 (35.60)	457 (48.16)
P-value			0.030*		0.005*		0.005*		0.61	
Age	6–11	463 (46.60)	7 (46.70)	456 (46.58)	12 (54.50)	451 (46.40)	12 (54.50)	451 (46.40)	22 (48.90)	441 (46.47)
	12–15	531 (53.40)	8 (53.33)	523 (53.42)	10 (45.50)	521 (53.60)	10 (45.50)	521 (53.60)	23 (51.10)	508 (53.53)
P-value			0.99		0.44		0.44		0.7507	
Grade level	1–4	561 (56.40)	8 (53.33)	553 (56.50)	13 (59.10)	548 (56.40)	13 (59.10)	548 (56.40)	24 (53.30)	537 (56.59)
	5–8	433 (43.60)	7 (46.67)	426 (43.50)	9 (41.90)	424 (43.60)	9 (41.90)	424 (43.60)	21 (46.70)	412(43.41)
P- value			0.8070		0.79		0.79		0.66	
Residence	Rural	765 (77.00)	13(86.70)	752 (76.80)	17 (77.27)	748 (77.00)	17 (77.27)	748 (77.00)	35 (77.80)	730 (77.00)
	Semi-urban	229 (23.00)	2 (13.30)	227 (23.20)	5 (22.73)	224 (23.00)	5 (22.73)	224 (23.00)	10 (22.20)	219 (23.00)
P- value			0.36		0.97		0.97		0.89	

+ *ve* positive, *-ve* negative, *qPCR* quantitative Polymerase Chain Reaction, *X*^*2*^*- test* Chi-squared test, *Total No.*: Total number

^*^Statistically significant if the P value for a chi-square test is less than 0.05

### The diagnostic performances of malaria RDTs

Using microscopy as a reference method, the diagnostic performances of the hsRDT were identical with the cRDT (Table 2). Accordingly, the sensitivity of the RDTs was 100% (95% CI 78.20, 100) and their specificity was 99.28% (95% CI 98.53, 99.71). It also demonstrated a PPV of 68.20 (50.62, 81.77) and an NPV of 100% (95% CI (99.60, 100). The RDTs also showed a strong agreement with results of microscopy at kappa value 81% (95% CI 67.00, 94.69). Thus, the overall probability of the RDTs to correctly classifying a school child with asymptomatic malaria (accuracy) was 99.30% (95% CI 98.55, 99.72).

**Table 2:** Diagnostic performance of malaria RDTs using microscopy as a reference

Malaria RDTs		Microscopy								
		+ ve	-ve	Total	Sensitivity (95% CI)	Specificity (95% CI)	PPV (95% CI)	NPV (95% CI)	Kapa (95% CI)	Accuracy (95% CI)
hsRDT	+ ve	15	7	22	100 (78.20, 100)	99.28 (98.53, 99.71)	68.20 (50.62, 81.77)	100 (99.60, 100)	81 (67.00, 94.69)	99.30 (98.55, 99.72)
	−ve	0	972	972						
Total		15	979	994						
cRDT	+ ve	15	7	22	100 (78.20, 100)	99.28 (98.53, 99.71)	68.20 (50.62, 81.77)	100 (99.60 100)	81 (67.00, 94.69)	99.30 (98.55, 99.72)
	−ve	0	972	972						
Total		15	979	994						

+ *ve* positive, *-ve* negative, *PPV* Positive predictive value, *NPV* Negative predictive value

Using qPCR as a reference method (Table 3). The sensitivity of the hsRDT was 48.89% (95% CI 33.70, 64.23) and that of the microscopy was 33% (95% CI 33.33 (20; 48.95)]. The hsRDT demonstrated similar PPV 100% (95% CI 85.00, 100) and specificity [100% (95% CI 99.61, 100)] with microscopy [specificity 100% (95% CI (99.61, 100) and PPV of 100% (95% CI 79.60, 100)]. In contrast, it achieved a better NPV [70.51% (95% CI 64.25; 76.09) than the microscopy [64.71% (95% CI 59.86; 69.27)]. The RDT showed good level of agreement with results of qPCR at kappa value 64% (95% CI 51.25, 77.99). The microscopy on the other hand showed moderate level of agreement with qPCR results [kappa = 49% (95% CI 33, 64.3). The overall probability that a school child with asymptomatic malaria was correctly classified (test accuracy) by the new kit was 74.4% (95% CI 74.26, 79.58) than microscopy [66.7% (95% CI 67.04, 72.84).

**Table 3:** Diagnostic performance of malaria RDTs using qPCR as a reference method

Malaria diagnostic tools		qPCR								
		+ ve	-ve	Total	Sensitivity (95% CI)	Specificity (95% CI)	PPV (95% CI)	NPV (95% CI)	Kapa (95% CI)	Accuracy (95% CI)
hsRDT	+ ve	22	0	22	48.89 (33.70, 64.23)	100 (99.61, 100)	100 (85.00, 100)	70.51 (64.25, 76.09)	64 (51.00, 78.00)	74.4 (74.26, 79.58)
	−ve	23	949	972						
Total		45	949	994						
cRDT	+ ve	22	0	22	48.89 (33.70, 64.23)	100 (99.61, 100)	100 (85.00, 100)	70.51 (64.25,76.09)	64 (51.00, 78.00)	74.4 (74.26, 79.58)
	−ve	23	949	972						
Total		45	949	994						
Microscopy	+ ve	15	0	15	33.33 (20, 48)	100 (99.61, 100)	100 (79.60,100)	64.71 (59.86,69.27)	49 (33.00, 64.30)	66.7 (67.04,72.84)
	−ve	30	949	979						
Total		45	949	994						

+ *ve* positive, *-ve* negative, *PPV* positive predictive value, *NPV* negative predictive value

### Prevalence of asymptomatic *P. falciparum* malaria in school children using qPCR, RDTs and microscopy

**Fig. 3:**
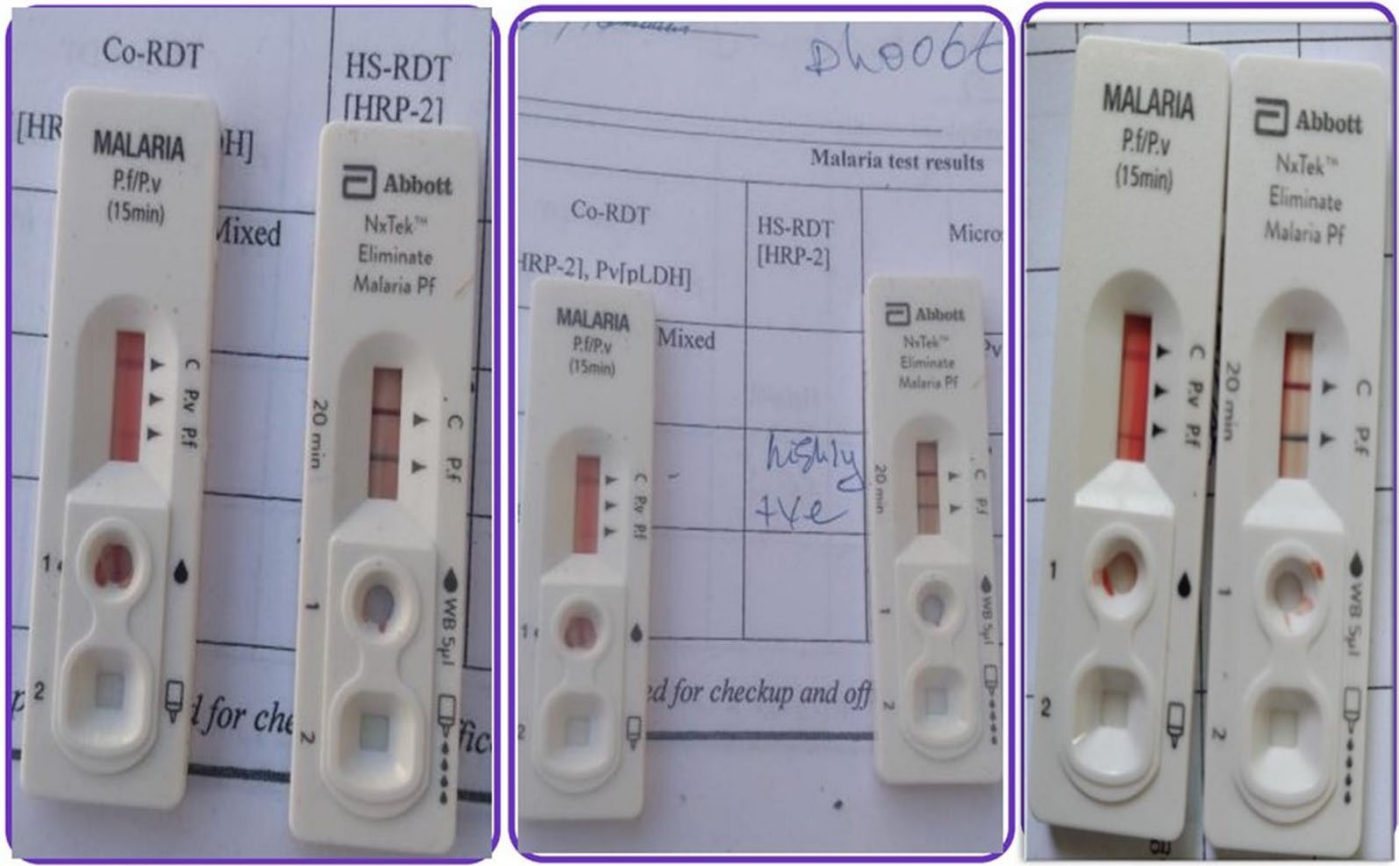
Plate 1 showing the differences in band line positivity of hsRDT and cRDT for detection of *P. falciparum HRP*2 from whole blood for detection of asymptomatic malaria of different study participants

Detection rates of asymptomatic *falciparum* malaria varied depending on the method used (Table 1). The overall prevalence of the infection was 4.52% (45/994) as determined by qPCR, of which 2.2% was detected by hsRDT/cRDT and 1.51% by microscopy. Across all malaria diagnostic tools, high infections were observed in males, grades 1–4, and children living in rural areas. There was statistically significant association between malaria positivity by conventional diagnostic tools (microscopy and RDTs) and sex of children (p < 0.0.05). Despite both RDTs had matching results, few cRDTs showed more fainter positive band lines than hsRDT (Fig. 3). On the other hand, the cRDT did not detect *P. vivax* in the current study population.

**Fig. 4:**
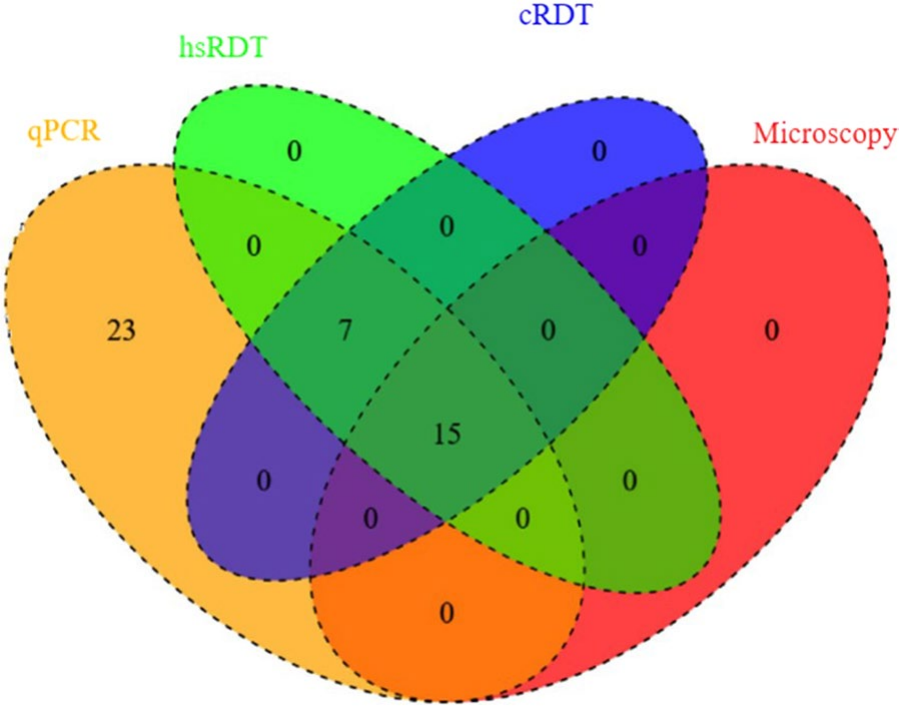
Venn diagram showing *P. falciparum* positivity intersections or unique to the malaria diagnostic tool(s) in school children with asymptomatic malaria infection

The Venn diagram (Fig. 4) shows a summary of the overall positive intersections of the malaria diagnostic methods used in the current study. Of the 22 cases identified by hsRDT, microscopy missed seven as false negatives. Likewise, 67% of the 45 cases detected by qPCR were submicroscopic malaria infections, as microscopy missed 30 of them as false negatives. On the other hand, the hsRDT- based detection shows a false negative rate of 51.1% (23/45). These results reflect the superior advantage of the hsRDT over microscopy for diagnosing *P. falciparum* malaria, and a silently ongoing transmission of the disease among children in the study area.

Asexual parasite density was determined using microscopic examination of Giemsa-stained thick blood smears using the microscope and ranged from 64 to 8080 parasites/µL and was divided into four categories (Table 4).

**Table 4:** Distribution of asymptomatic *P. falciparum density* by sex, age group, grade level, and place of residence

Participants n (%)									
Parasite density/μL	N	Sex		Age group		Grade level		Residence	
		Male	Female	6–11	12–15	1–4	5–8	Rural	Semi-urban
< 200	5	3 (25%)	2 (67%)	2 (29%)	3 (38%)	2 (25%)	3 (43%)	3 (23%)	2 (100%)
201–200	3	2 (17%)	1 (33%)	1 (14%)	2 (25%)	1 (13%)	2 (29%)	3 (23%)	0 (0%)
501–2000	3	3 (25%)	0 (0%)	2 (29%)	1 (13%)	2 (25%)	1 (14%)	3 (23%)	0 (0%)
2001–9000	4	4 (33%)	0 (0%)	2 (29%)	2 (25%)	3 (38%)	1 (14%)	4 (31%)	0 (0%)

*N* number

## Discussion

As the Global Technical Strategy on Malaria 2016–2030 envisions a malaria-free world by 2030 with the aim of a 90% reduction in the global malaria burden [31] is approaching, the role of HRP2-based malaria RDT is paramount in monitoring and steering the success of the Strategy. The current study evaluated the field performance of the hsRDT for the detection of *P. falciparum* HRP2 antigen in whole blood from healthy school children with asymptomatic malaria. The results of this study confirmed the high sensitivity (100%) and specificity (99.28%) of hsRDT using microscopy as a reference method, which is consistent with the WHO prequalification report [21]. However, the study participants in the report were symptomatic patients, and venipuncture blood was used for the analysis, as opposed to finger-prick blood samples collected from healthy school children in this study. The higher PPV (96.1%) and NPV (100%) in this this study is comparable with the study conducted in Saudi Arabia [32] and Kenya [33].

When qPCR was used as a reference, the hsRDT had sensitivity (48.89%) than that of microscopy (33.3%) and same specificity and PPV (100%). It also demonstrated a higher NPV (70.51%) and accuracy (74.4%) in correctly classifying children with asymptomatic malaria infection. The lower sensitivity in the current study is compared to the manufacturer's report [21, 22, 26] though they used a composite sensitivity (microscopy + qPCR). The difference could be attributed to variations in study population, source of specimen, and disease status. The sensitivity of hsRDT is also lower compared to a study conducted in western Kenya (60.3%) in asymptomatic school-age children [34]. However, higher specificity and PPV were observed in the current study. The sensitivity of hsRDT in this study is also lower compared to a recently reported meta-analysis which estimated a pooled sensitivity at 56.1% in asymptomatic population and higher than that of cRDT (44.3%) in the report [20]. A similar performance of the hsRDT as cRDT in this study is consistent with those from Mozambique and Uganda [35] where the diagnostic performances of ultrasensitive RDT (uRDT) and cRDT are comparable. A study from Uganda and Myanmar [36] using samples from the general population with asymptomatic malaria reported that hsRDT showed a tenfold improvement in detecting HRP2 compared to the cRDT. On the other hand, Landier and colleagues [37] in East Myanmar reported that the sensitivity of hsRDT was two-fold higher than that of cRDT. The sensitivity of hsRDT in our study is slightly higher than that performed in a low-transmission setting in Myanmar (44%) and lower than that performed in a high-transmission setting in Ugandan children (84%). However, the new kit showed higher specificity (100%) than the Uganda report (95%) and is consistent with a report from Myanmar. Interestingly, the same study reported that the cRDT had a sensitivity of 62% in Uganda and 0% in Myanmar [36]. Existing evidences support these contradicting results, that might be attributed to differences in type and sensitivity of qPCR sensitivity [38] and parasite density and transmission settings [39]. It is also important to recall that, mosquito bites are usually rare in low transmission settings, so that the parasite's main strategy is successful transmission with only a few bites, while the it becomes non-virulent [40, 41]. However, in high transmission settings, the mosquito bite is so frequent and the priority of the parasite is surmounting the host immunity [41].

The outperformance of the new kit over the established method suggests that it can be considered a replacement for microscopy in a resource-poor rural setting. The results of the current study confirmed that the new kit offers no added value over the existing malaria RDT in the study population. However, available evidences indicate that the performance of HRP2- based malaria RDTs could be affected by the parasite density [42, 43], HRP2/3 -gene deletions [44] and the prozone effect in PfHRP2 [45].

Therefore, considering these points, in this study, the real comparison between the RDTs could have been compromised by all or any of these factors. In general, given that the cost and performance of the cRDT is comparable, the hsRDT can be a useful tool to diagnose asymptomatic malaria as part of the national malaria elimination programme.

In the current study, the proportion of infections detected by the RDTs was the same (2.2%, 22/994), higher than microscopy (1.5%, 15/994). Infection was higher in male school children and those from rural areas. This could possibly be due to male boys being more likely to play outdoors than female girls, and more mosquito bites in rural than urban areas. The proportion of detection of infection by RDTs [2.21% (22/994)] in this study is lower than a study conducted in Uganda [22] in which the hsRDT detected 24.9% (546/2,192) in school-age children. A closer look at this data show that microscopy missed 31.8% (7/22) of the infected children identified by RDTs, confirming the diagnostic advantage of the RDTs over microscopy in detecting low-density malaria infections. Likewise, microscopy missed 66.7% (30/45) of infected children identified by qPCR. This reveals that asymptomatic submicroscopic falciparum malaria infection was prevalent among school children in the study area. This is reinforced by previous work by Okell and colleagues [9] who reported that microscopy misses 20–50% of malaria infections in areas of low transmission and is responsible for all human-to-mosquito transmission. Hence, the results of this study challenge the importance of light microscopy for malaria control and elimination. Likewise, both RDTs missed 51% (23/45) of infected children identified by qPCR. As explained above, this RDT-negative/PCR-positive samples could be partially explained as (1) possible prevalence of HRP2/3-gene deletions in most samples or (2) due to low concentration of circulating HRP2 antigens which could not be detected and result in false-negative results [44] and in individuals with rheumatoid arthritis, cross-reactivity of rheumatoid factors with HRP2 results in false-positive results [46]. At this point it should be noted that the conventional tool-based diagnosis underestimates the actual prevalence of falciparum malaria in the study population. In all cases, it was shown that there was silent and active transmission of the infection in the area.

Although the performance of both RDTs were identical in this study**,** differences in the intensity of the HRP2 positive band lines were observed. For example, some cRDT tests showed very faint positive Pf band lines that was difficult to declare, but all were clearly visible with the new RDT**.** These differences could be attributed either to the assumption of a low concentration of circulating HRP2 antigen in asymptomatic school children or the fluoresce material used. Briefly, both tests are lateral flow immunochromatographic kits that allow for a qualitative diagnosis based on the detection of HRP2 expressed by *P. falciparum* in the blood of infected individuals [47]. Available evidence shows that the limit of detection (LOD) for HRP2 by most cRDTs is estimated at 800–1000 pg/mL when the parasitaemia is 100–200/µL [48] and the visibility of bands diminishes when the parasitaemia falls below 200 p/µL [49], indicating that parasite density and HRP2 concentration are directly related.

Another plausible piece of evidence in support of this observations is a study on the analytical performance of hsRDT [50], which demonstrated better detection of HRP2 at a tenfold lower LOD than most RDTs, suggesting that the new kit detects HRP2 better than cRDT in low-density parasitaemia. So far, there are only very few empirically published reports examining the HRP2 concentration and LOD of cRDT and HSRDT. For example, Jimenez and colleagues [48] compared both RDTs for their performance on samples with known HRP2 concentrations. The authors reported that the LOD of cRDT was 800 pg/L compared to 8 pg/L LOD for uRDT. Furthermore, in this study, the true comparison between both RDTs might have been compromised by the presence of HRP2 gene deletions in most of the samples, as the qPCR result shows.

## Conclusions

The current study reports the diagnostic performance of the NxTek^™^ Elimination Malaria Pf Ag test in detecting *P. falciparum* in school children with asymptomatic malaria in Ethiopia. The hsRDT detected 7- fold more low-density *P. falciparum* malaria infections and provides a better measure of discriminating children with and without asymptomatic malaria (diagnostic value) than the malaria gold standard tool, microscopy. On the contrary, the new kit did not outperform the conventional RDT (SD Bioline Malaria Ag Pf. P.v) in the current study. The study provides significant numbers of asymptomatic submicroscopic *P. falciparum* infections. This active and silent transmission of malaria in school-age children is an alarm for the malaria control programme in the Gomma District of the Jima Zone for appropriate interventions. The results of the current study also underscore the importance of molecular tools for screening asymptomatic populations to achieve malaria elimination goals.

## Data Availability

The datasets used in the current study are available from the corresponding author upon request.

## References

[1] WHO (2021). World malaria report 2021.

[2] Holmes KK, Bertozzi S, Bloom BR, Jha P, Gelband H, DeMaria LM, Holmes KK, Bertozzi S, Bloom BR, Jha P (2017). Major infectious diseases: key messages from disease control priorities. Major infectious diseases.

[3] Federal Democratic Republic of Ethiopia Ministry of Health (2017). National malaria elimination roadmap.

[4] Cohee LM, Nankabirwa JI, Greenwood B, Djimde A, Mathanga DP (2021). Time for malaria control in school-age children. Lancet Child Adolesc Health.

[5] Cohee LM, Valim C, Coalson JE, Nyambalo A, Chilombe M, Ngwira A (2021). School-based screening and treatment may reduce *P. falciparum* transmission. Sci Rep.

[6] Ashton RA, Kefyalew T, Tesfaye G, Pullan PL, Yadeta D, Reithinger R (2011). School-based surveys of malaria in Oromia Regional State, Ethiopia: a rapid survey method for malaria in low transmission settings. Malar J.

[7] Federal Democratic Republic of Ethiopia Ministry of Health (2020). National malaria elimination strategic plan: 2021–2025.

[8] Whittaker C, Slater H, Nash R, Bousema T, Drakeley C, Ghani AC (2021). Global patterns of submicroscopic *Plasmodium falciparum* malaria infection: insights from a systematic review and meta-analysis of population surveys. Lancet Microbe.

[9] Okell LC, Bousema T, Griffin JT, Ouédraogo AL, Ghani AC, Drakeley CJ (2012). Factors determining the occurrence of submicroscopic malaria infections and their relevance for control. Nat Commun.

[10] Chen I, Clarke SE, Gosling R, Hamainza B, Killeen G, Magill A (2016). “Asymptomatic” malaria: a chronic and debilitating infection that should be treated. PLoS Med.

[11] Notomi T, Okayama H, Masubuchi H, Yonekawa T, Watanabe K, Amino N (2000). Loop-mediated isothermal amplification of DNA. Nucleic Acids Res.

[12] Hopkins H, González IJ, Polley SD, Angutoko P, Ategeka J, Asiimwe C (2013). Highly sensitive detection of malaria parasitemia in a malaria-endemic setting: performance of a new loop-mediated isothermal amplification kit in a remote clinic in Uganda. J Infect Dis.

[13] Golassa L, Messele A, Amambua-Ngwa A, Swedberg G (2020). High prevalence and extended deletions in *Plasmodium falciparum* hrp2/3 genomic loci in Ethiopia. PLoS ONE.

[14] Menegon M, L'Episcopia M, Nurahmed AM, Talha AA, Nour BYM, Severini C (2017). Identification of *Plasmodium falciparum* isolates lacking histidine-rich protein 2 and 3 in Eritrea. Infect Genet Evol.

[15] Berhane A, Anderson K, Mihreteab S, Gresty K, Rogier E, Mohamed S (2018). Major threat to malaria control programes by *Plasmodium falciparum* lacking histidine rich protein 2. Eritrea Emerg Infect Dis.

[16] Tedla M (2019). A focus on improving molecular diagnostic approaches to malaria control and elimination in low transmission settings. Parasite Epidemiol Control.

[17] Britton S, Cheng Q, McCarthy JS (2016). Novel molecular diagnostic tools for malaria elimination: a review of options from the point of view of high-throughput and applicability in resource limited settings. Malar J.

[18] Cheaveau J, Mogollon DC, Mohon MAN, Golassa L, Yewhalaw D, Pillai DR (2019). Asymptomatic malaria in the clinical and public health context. Expert Rev Anti Infect Ther.

[19] Feachem RG, Chen I, Akbari O, Bertozzi-Villa A, Bhatt S, Binka F (2019). Malaria eradication within a generation: ambitious, achievable, and necessary. Lancet.

[20] Slater HC, Ding XC, Knudson S, Bridges DJ, Moonga H, Saad NJ (2022). Performance and utility of more highly sensitive malaria rapid diagnostic tests. BMC Infect Dis.

[21] WHO (2020). Prequalification of in vitro diagnostics; public report. NxTek Eliminate Malaria Pf. WHO reference number: PQDx 0349–012-00.

[22] Kyabayinze D, Kajungu D, Nambuya P, Okiira C, Ndawula B, Kawooya M (2021). Evaluating field performance of highly sensitive malaria RDT: detection of infection among febrile patients, asymptomatic pregnant women and household contacts in Mpigi, Uganda.

[23] Worku L, Damtie D, Endris M, Getie S, Aemero M (2014). Asymptomatic malaria and associated risk factors among school children in Sanja town, Northwest Ethiopia. Int Sch Res Notices.

[24] Brooker S, Kolaczinski JH, Gitonga CW, Noor AM, Snow RW (2009). The use of schools for malaria surveillance and programme evaluation in Africa. Malar J.

[25] Gitonga CWKJ, Njenga SM, Awuondo K, Noor AM, Snow RW (2012). Use of rapid diagnostic tests in malaria school surveys in Kenya: does their under-performance matter for planning malaria control?. Am J Trop Med Hyg.

[26] Abbott Diagnostics Korea Inc. NxTek™ Eliminate Malaria P.f. https://www.globalpointofcare.abbott/en/product-details/nxtek-eliminate-malaria-pf.html.2021 Accessed 17 Mar 2021.

[27] WHO (2016). Malaria microscopy standard operating procedure—MM-SOP-09.

[28] Wooden J, Kyes S, Sibley C (1993). PCR and strain identification in *Plasmodium falciparum*. Parasitol Today.

[29] Rosanas-Urgell A, Mueller D, Betuela I, Barnadas C, Iga J, Zimmerman PA (2010). Comparison of diagnostic methods for the detection and quantification of the four sympatric *Plasmodium* species in field samples from Papua New Guinea. Malar J.

[30] Veron V, Simon S, Carme B (2009). Multiplex real-time PCR detection of *P. falciparum*, *P. vivax* and *P. malariae* in human blood samples. Exp Parasitol.

[31] WHO (2021). Global technical strategy for malaria 2016–2030 (2021 update).

[32] Madkhali AM, Ghzwani AH, Al-Mekhlafi HM (2022). Comparison of rapid diagnostic test, microscopy, and polymerase chain reaction for the detection of *Plasmodium falciparum* malaria in a low-transmission area, Jazan Region. Southwestern Saudi Arabia Diagnostics.

[33] Wanja EW, Kuya N, Moranga C, Hickman M, Johnson JD, Moseti C (2016). Field evaluation of diagnostic performance of malaria rapid diagnostic tests in western Kenya. Malar J.

[34] Turnbull LB, Ayodo G, Knight V, John CC, McHenry MS, Tran TM (2022). Evaluation of an ultrasensitive HRP2–based rapid diagnostic test for detection of asymptomatic *Plasmodium falciparum* parasitaemia among children in western Kenya. Malar J.

[35] Owalla TJ, Okurut E, Apungia G, Ojakol B, Lema J, Murphy SC (2020). Using the ultrasensitive Alere *Plasmodium falciparum* Malaria Ag HRP-2™ rapid diagnostic test in the field and clinic in Northeastern Uganda. Am J Trop Med Hyg.

[36] Das S, Jang IK, Barney B, Peck R, Rek JC, Arinaitwe E (2017). Performance of a high-sensitivity rapid diagnostic test for *Plasmodium falciparum* malaria in asymptomatic individuals from Uganda and Myanmar and naive human challenge infections. Am J Trop Med Hyg.

[37] Landier J, Haohankhunnatham W, Das S, Konghahong K, Christensen P, Raksuansak J (2018). Operational performance of a *Plasmodium falciparum* ultrasensitive rapid diagnostic test for detection of asymptomatic infections in Eastern Myanmar. J Clin Microbiol.

[38] Gruenberg M, Moniz CA, Hofmann NE, Koepfli C, Robinson LJ, Nate E (2020). Utility of ultra-sensitive qPCR to detect *Plasmodium falciparum* and *Plasmodium vivax* infections under different transmission intensities. Malar J.

[39] Hofmann NE, Gruenberg M, Nate E, Ura A, Rodriguez-Rodriguez D, Salib M (2018). Assessment of ultra-sensitive malaria diagnosis versus standard molecular diagnostics for malaria elimination: an in-depth molecular community cross-sectional study. Lancet Infect Dis.

[40] Mackinnon MJ, Read AF (2004). Virulence in malaria: an evolutionary viewpoint. Philos Trans R Soc Lond Biol Sci.

[41] Rono MK, Nyonda MA, Simam JJ, Ngoi JM, Mok S, Kortok MM (2018). Adaptation of *Plasmodium falciparum* to its transmission environment. Nat Ecol Evol.

[42] Kojom Foko LP, Pande V, Singh V (2021). Field performances of rapid diagnostic tests detecting human *Plasmodium* species: a systematic review and meta-analysis in India, 1990–2020. Diagnostics.

[43] Singh N, Bharti PK, Singh MP, Mishra S, Shukla MM, Sharma RK (2013). Comparative evaluation of bivalent malaria rapid diagnostic tests versus traditional methods in field with special reference to heat stability testing in central India. PLoS ONE.

[44] Feleke SM, Reichert EN, Mohammed H, Brhane BG, Mekete K, Mamo H (2021). *Plasmodium falciparum* is evolving to escape malaria rapid diagnostic tests in Ethiopia. Nat Microbiol.

[45] Gillet P, Scheirlinck A, Stokx J, De Weggheleire A, Chaúque HS, Canhanga OD (2011). Prozone in malaria rapid diagnostics tests: how many cases are missed?. Malar J.

[46] Iqbal J, Sher A, Rab A (2000). *Plasmodium falciparum* histidine-rich protein 2-based immunocapture diagnostic assay for malaria: cross-reactivity with rheumatoid factors. J Clin Microbiol.

[47] Cunningham J, Jones S, Gatton ML, Barnwell JW, Cheng Q, Chiodini PL (2019). A review of the WHO malaria rapid diagnostic test product testing programme (2008–2018): performance, procurement and policy. Malar J.

[48] Jimenez A, Rees-Channer RR, Perera R, Gamboa D, Chiodini PL, González IJ (2017). Analytical sensitivity of current best-in-class malaria rapid diagnostic tests. Malar J.

[49] Ishengoma DS, Francis F, Mmbando BP, Lusingu J, Magistrado P, Alifrangis M (2011). Accuracy of malaria rapid diagnostic tests in community studies and their impact on treatment of malaria in an area with declining malaria burden in north-eastern Tanzania. Malar J.

[50] Das S, Peck RB, Barney R, Jang IK, Kahn M, Zhu M (2018). Performance of an ultra-sensitive *Plasmodium falciparum* HRP2-based rapid diagnostic test with recombinant HRP2, culture parasites, and archived whole blood samples. Malar J.

